# Supplementary material for: Synovial sarcoma reprograms transcription by GBAF activation of polycomb targets and loss of CBAF enhancers
Source: Nat Commun. 2025 Dec 21;17:1081. doi: 10.1038/s41467-025-67841-y (PMC12852873; doi:10.1038/s41467-025-67841-y)
Supplement: Supplementary file 1 — Supplementary Information [file 41467_2025_67841_MOESM1_ESM.pdf]

Supplementary Figures with Legends

Synovial sarcoma reprograms transcription by GBAF activation of polycomb targets and loss of CBAF enhancers

Jinxiu Li, Li Li, Kyllie Smith-Fry, Muhammad Zaki Fadlullah, Lara Carroll, Linda Morrison, Xinyi Ge, Mary Nelson, Lesley A. Hill, Yixuan Guo, George Davenport, Xiaoyang Zhang, Torsten O. Nielsen, Martin Hirst, T. Michael Underhill, Bradley R. Cairns, Kevin B. Jones

Supplementary Table 1. Key resources and materials.

Reagent or resource	SOURCE	IDENTIFIER
**Antibodies**		
Anti-SMARCA4 antibody (Western)	Abcam	Cat# ab4081, RRID:AB_304271
Anti-SMARCA4 antibody (ChIP)	Abcam	Cat# ab110641, RRID:AB_10861578
Anti-SMARCB1 antibody (Western)	Bethyl	Cat# A301-087A, RRID:AB_2191714
Anti-SMARCC1 antibody (Western)	Proteintech	Cat# 17722-1-AP, RRID:AB_2191987
Anti-SMARCC1 antibody (ChIP)	Invitrogen	Cat# PA5-30174, RRID:AB_2547648
Anti-SS18 antibody (Western)	Cell Signaling	Cat# 21792, RRID:AB_2728667
Anti-SS18-SSX antibody (Western and ChIP)	Cell Signaling	Cat# 72364
Anti-PBRM1 antibody (Western)	Bethyl	Cat# A301-591A, RRID:AB_1078808
Anti-PBRM1 antibody (ChIP)	Cell Signaling	Cat# 89123, RRID:AB_2936366
Anti-ARIAD1A antibody (Western, ChIP)	Cell Signaling	Cat# 12354, RRID:AB_2637010
Anti-BRD9 antibody (Western and ChIP)	Proteintech	24785-1-AP
Anti-DPF2 antibody (Western and ChIP)	Abcam	Cat# ab134942, RRID:AB_2728668
H3K4me1 (ChIP)	Diagenode	Cat# C15410037-50, CiteAb: pAb-037-050
H3K4me3 (ChIP)	Cell Signaling	Cat# 9751S, RRID:AB_2616028
H3K27ac (ChIP and HiChIP)	Abcam	Cat# ab4729, RRID:AB_2118291
H3K27me3 (ChIP)	Diagenode	Cat# C15410069, CiteAb: pAb-069-050
H3K36me3 (ChIP)	Abcam	Cat# ab9050, RRID:AB_306966
H2AK119ub (ChIP)	Cell Signaling	Cat# 8240T, RRID:AB_10891618
Goat Anti-Rabbit IgG (H + L)-HRP Conjugate	BIO-RAD	Cat# 170-6515, RRID:AB_11125142

Goat Anti-Mouse IgG (H + L)-HRP Conjugate	BIO-RAD	Cat# 170-6516, RRID:AB_11125547
SOX2 (IF)	Abcam	Cat# ab97959, RRID:AB_2341193
TLE1 (IF)	Abcam	ab183742
Goat Anti-Rat IgG (H+L) Antibody, Alexa Fluor™ 647 Conjugated (IF)	Invitrogen	Cat# A-21247, RRID:AB_141778
Goat Anti-Rabbit IgG (H+L) Antibody, Alexa Fluor™ 594 Conjugated (IF)	Invitrogen	Cat# A-11012, RRID:AB_141359
Goat anti-Chicken IgY (H+L) Secondary Antibody, Alexa Fluor™ 488 (IF)	Invitrogen	Cat# A-11039, RRID:AB_2534096
GFP (IF)	Abcam	Cat# ab13970, RRID:AB_300798
**Chemicals, Peptides, and Recombinant Proteins**		
HSBB	Gibco	14025092
PBS, pH 7.4	Gibco	10010023
Bovine Serum Albumin (BSA) DNase- and Protease-free Powder	Fisher Scientific	BP9706100
Paraformaldehyde 16% (w/v) in aqueous solution Methanol free	Alfa Aesar	43368
Trizma® base	Sigma-Aldrich	T6066
Glycine	Sigma-Aldrich	G8898
TT1	Covaris	520001
TT1 Adapter milliTUBE	Covaris	520142
TC13 Tube	Covaris	520010
Protease Inhibitor Cocktail	Sigma-Aldrich	P8340-5ML
Dynabeads™ M-280 Sheep Anti-Rabbit IgG	Invitrogen	11204D
streptavidin C1	Invitrogen	65001
Power SYBR™ Green PCR Master Mix	Applied Biosystems	4367659
Thinwall Polypropylene Tubes, 14 x 89mm	Beckman	331372

SuperSignal™ West Dura Extended Duration Substrate	Thermo Scientific	34076
2-Mercaptoethanol	Sigma-Aldrich	M3148
30% Acrylamide/Bis Solution, 37.5:1	BIO-RAD	1610158
Proteinase K	Invitrogen	25530049
Cre Recombinase	Excellgen	EG-1001
MboI restriction enzyme	NEB	R0147M
biotin-dATP	Invitrogen	19524016
DNA Polymerase I, Large (Klenow) Fragment	NEB	M0210L
T4 DNA ligase	NEB	M0202
streptavidin C1	Invitrogen	65001
**Critical Commercial Assays**		
Direct-zolTM RNA Miniprep kit	Zymo	R2050
DNA Clean & Concentrator-5	Zymo	D4004
NEBNext Ultra II Directional RNA Library Prep Kit for Illumina	NEB	E7760L
NEBNext Ultra II DNA Library Prep kit	NEB	E7634L
Next GEM Single Cell 3′ Kit v3.1	10x Genomics	PN-1000268
NovaSeq X Series 10B Reagent Kit	Ilumina	20085594
**Deposited data**		
This work (ChIP-Seq, Hi-ChIP)	NIH GEO	GSE269770
This work (RNA-Seq)	NIH GEO	GSE269772
This work (scRNA-Seq)	NIH GEO	GSE269773

Software and Algorithms		
imageJ	Open Source/National Institutes of Health	https://imagej.nih.gov/ij/
GraphPad Prism softeware	GraphPad Software, Inc.	https://www.graphpad.com/scientific- software/prism/
Adobe Illustrator	Adobe	http://www.adobe.com/products/ illustrator.html
igv	Broad Institute	http://software.broadinstitute.org/software/igv/
deepTools	Max Planck Institute for Immunobiology and Epigenetics, Freiburg.	https://github.com/deeptools/deepTools/blob/develop/docs/index.rst
bedtools	Quinlan laboratory at the University of Utah	https://github.com/arq5x/bedtools2
UCSC Genome Browse	UCSC	http://genome.ucsc.edu/

Other	
mgHES1-F	CCTGGCACCCGTATCACAAA
mgHES1-R	AGTTGTACCCTGGCAGAGAGA
mgCCND1-F	ACACGCAAGCCAAGGAAGAA
mgCCND1-R	GCACCCATTCTCCCGGTTTA
mgCCND2-F	ACCATGTGGATGAGTTCAGATAACT
mgCCND2-R	GGTTTGGCGAGGTTGCAATT
mgSOX2-F	CTTGGGTCTAACTTCTCGTCTG
mgSOX2-R	GTGGTGTGCCATTGTTTCTG
Smarcb1 F	CACCATGCCCCCACCTCCCCTACA
Smarcb1 R	CAGGAAAATGGATGCAACTAAGAT
Arid1a F	TGGGCAGGAAAGAGTAATGG
Arid1a R	AACACCACTTTCCCATAGGC
Arid1b F	CTTGGTCTTACCCATTTGCAC
Arid1b R	ATCGATGGAGCCAGACAGGT
Pbrm1 F	GACATGGCTTCTCCCAAACT
Pbrm1 R	TGCAACTCTTTGTCCTTACACG
ROSA-WS268	GTTATCAGTAAGGGAGCTGCAGTGG
ROSA-WS270	AAGACCGCGAAGAGTTTGTCCTC
ROSA-WS271	GGCGGATCACAAGCAATAATAACC

Supplementary Fig. 1 (associated with Fig. 2): SS18::SSX distributes to the transcriptional regulatory elements of monoubiquitylated histone H2A bearing nucleosomes.

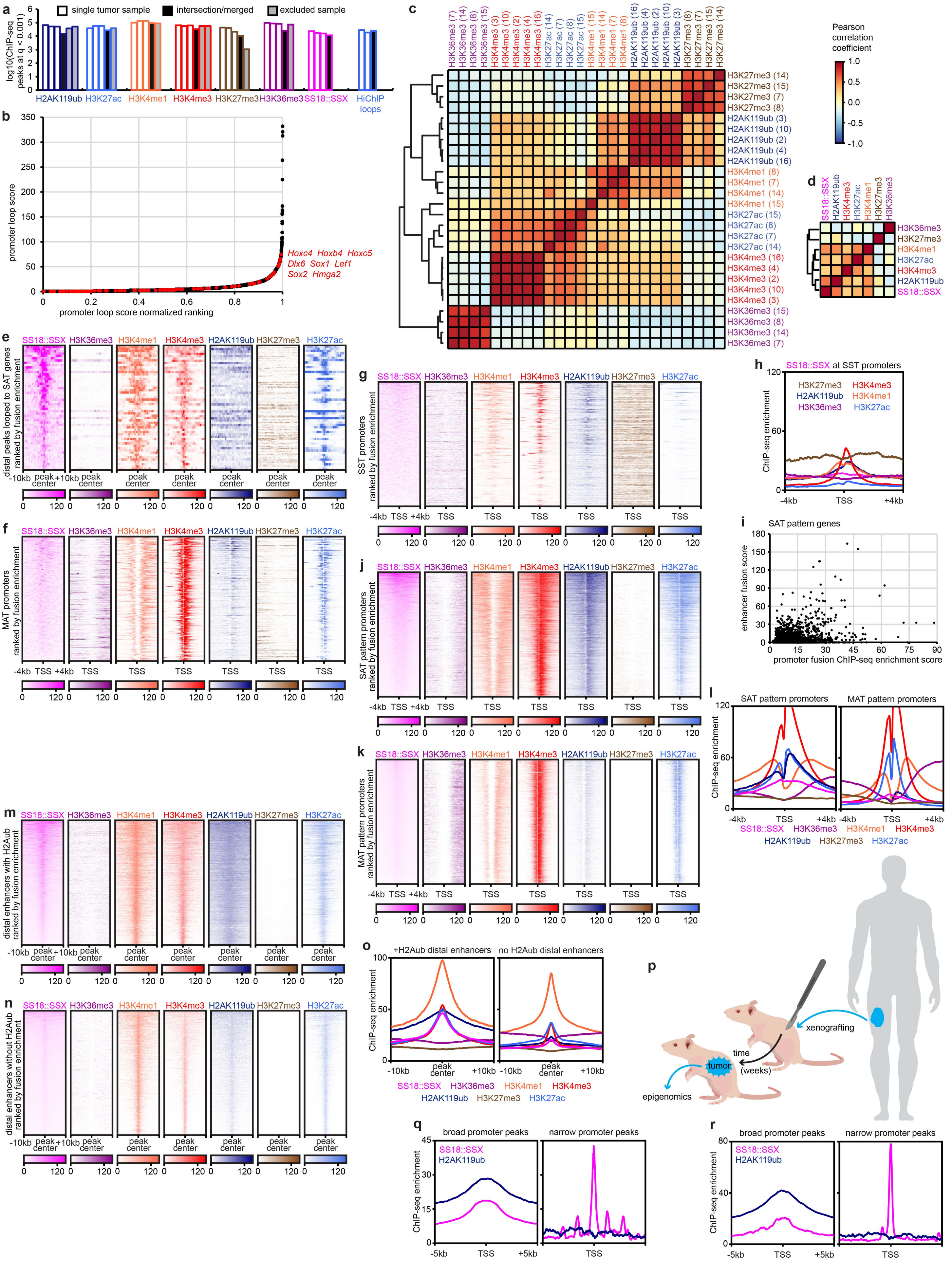

a Peak counts after calling in MACS2 with q-value < 0.001, noting which three of each antibody's ChIP-seq were intersected for a merged ChIP-seq peaks number shown with black filling, as well as any not included other samples. Also shown is the number of loops called for two tumors in H3K27ac HiChIP at FDR < 0.01 in Mango, then merged. **b** The H3K27ac HiChIP loops score for each looped promoter in the genome reflecting both score and a normalized ranking of the score, with SAT genes indicated in red. **c** Pearson correlation heatmap of individual samples of ChIP-seq for each indicated antibody, with the tumor numbers noted in parentheses. **d** Pearson correlation heatmap of intersection samples of each histone mark ChIP-seq with the fusion. **e** ChIP-seq enrichment heatmaps at distal fusion peaks looped to SAT gene promoters. **f** ChIP-seq enrichment heatmaps for the promoters of MAT genes. **g** ChIP-seq enrichment heatmaps and plots **h** for synovial sarcomagenesis silenced transcription genes. **i** Plot of looped distal fusion enrichment against promoter enrichment for all genes with SAT patterns in their promoters (or called peaks for $H3K4me3^{+}$, $H3K27ac^{+}$, H2AK119ub+, and not $H3K27me3^{-}$). **j** ChIP-seq enrichment heatmaps for SAT pattern promoters and MAT pattern promoters, **k**, as well as enrichment plots for both, **l**. **m** ChIP-seq enrichment heatmaps for distal enhancers with called peaks for H3K4me1, H3K27ac, and H2AK119ub versus the same **n** for those lacking H2AK119ub called peaks and **o** enrichment plots for each. **p** Schematic of patient derived xenografting (PDXing) of human SySs grown in immunocompromised mice to develop sizeable samples of tumors for bulk epigenomics. **q** SS18::SSX and H2AK119ub ChIP-seq enrichment plots for broad and narrow promoter fusion peaks in a PDX. **r** The same in a second PDX.

Supplementary Fig. 2 (associated with Fig. 3): BAF family complex subtypes distribute across chromatin relative to SS18::SSX.

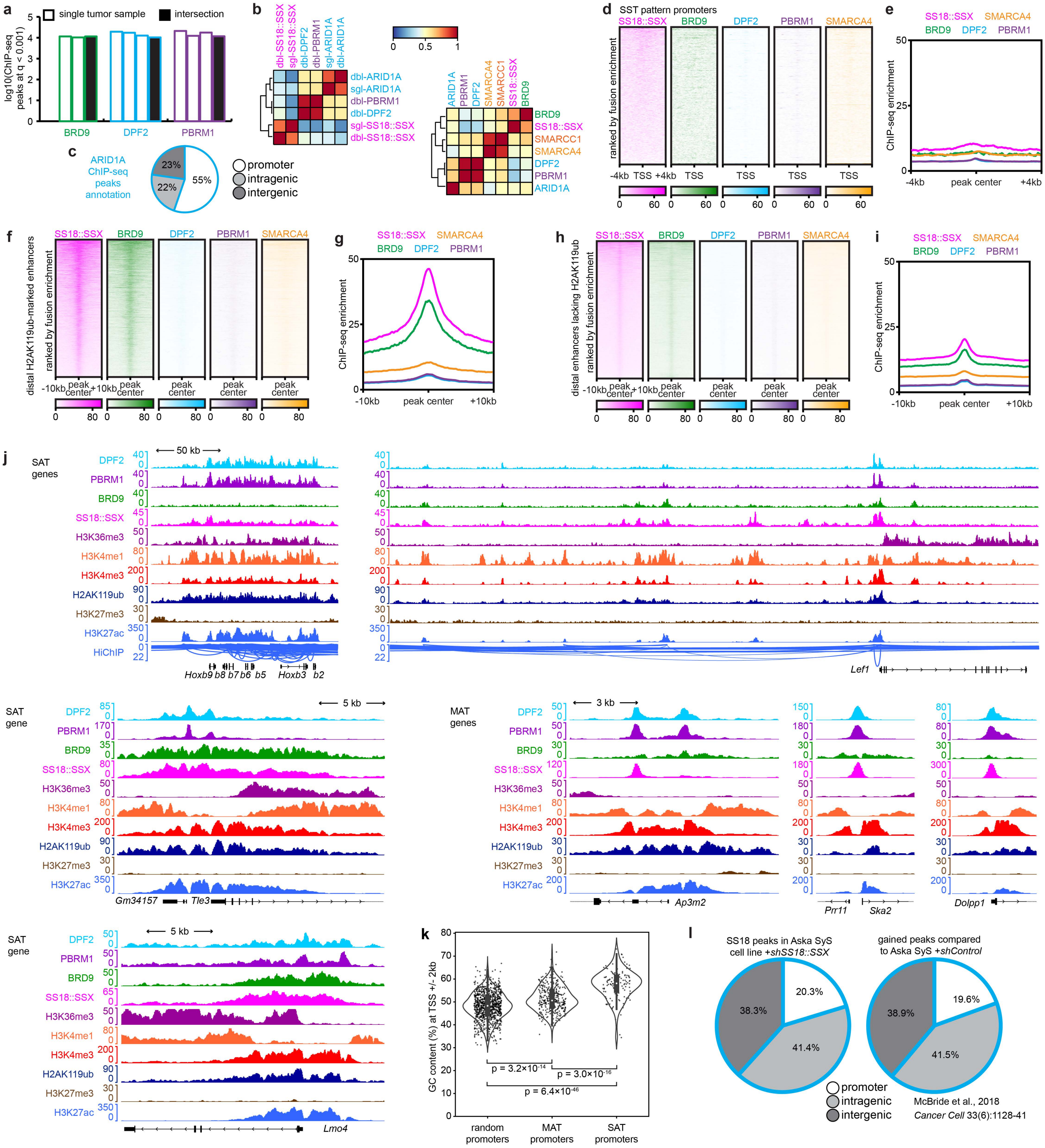

a Peak counts called for BAF component ChIP-seq in individual samples, and the intersection merged peaks set. **b** Pearson correlation heatmap for (left) individual ChIP-seq runs for the indicated antibodies after either single (sgl) or double (dbl) crosslinking and (right) intersection merged ChIP-seq for the indicated antibodies. **c** Annotation distribution of ARID1A ChIP-seq peaks, also demonstrating a predominance of promoter proximal peaks for CBAF. **d** ChIP-seq enrichment heatmaps for BAF components at SST gene promoters and **e** plots of the same. **f** ChIP-seq enrichment heatmaps and **g** plots for distal peaks that were determined by called peaks for H3K4me1, H3K27ac, and H2AK119ub, as well as those **h**,**i** lacking a called peak for H2AK119ub. **j** Example ChIP-seq enrichment tracks for SAT pattern genes with and without significant distal looped enhancers, as well as MAT genes. **k** Violin plot of the GC content in promoters of each pattern (Violin plots show data distribution with embedded box plots indicating median, 25th–75th percentiles, and whiskers for minimum and maximum. Sample sizes: Random sites, n = 977; MAT sites, n = 339; SAT sites, n = 126. Kruskal-Wallis test for unbalanced data, rank-based; p-value from Dunn test for pairwise comparisons). **l** Annotation distribution of SS18 ChIP-seq peaks after fusion depletion in the Aska human SyS cell line (representing CBAF peaks, generally) as well as the annotation distribution of gained peaks from control shRNA.

Supplemental Figure 3 (associated with Fig. 4). Single cell transcriptomics reveal distinct cell type clusters within synovial sarcoma tumors.

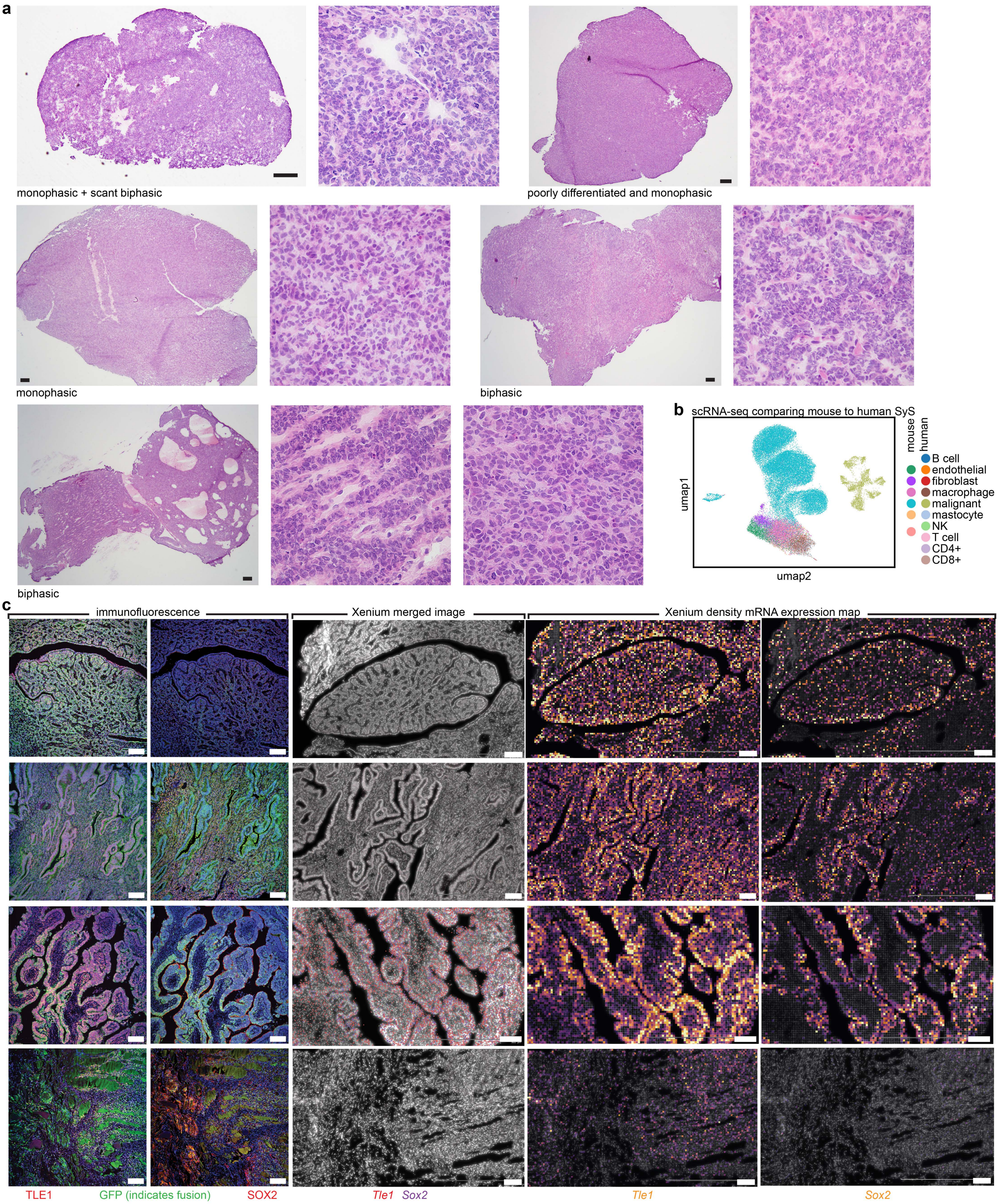

a Representative photomicrographs showing histomorphology of snap frozen tissue sections adjacent to areas sampled by single cell RNA-seq (Magnification bars, 250µm; square panel sides, 250µm. Note that snap freezing creates artifacts resembling nuclear atypia.) **b** correlation of mouse SyS scRNA-seq with human scRNA-seq using data and SyS gene list reported by Jerby-Arnon et al. *Nat Med*. 2021 Feb;27(2):289-300. **c** Immunofluorescence with antibodies against proteins coded for by two highly expressed SAT genes, TLE1 and SOX2 are marked to correlate with the Xenium spatial resolution transcriptomics (Magnification bars, 100µm).

Supplementary Fig. 4 (associated with Fig. 4): Xenium spatial transcriptomics identifies cell markers for each synovial sarcoma tissue type.

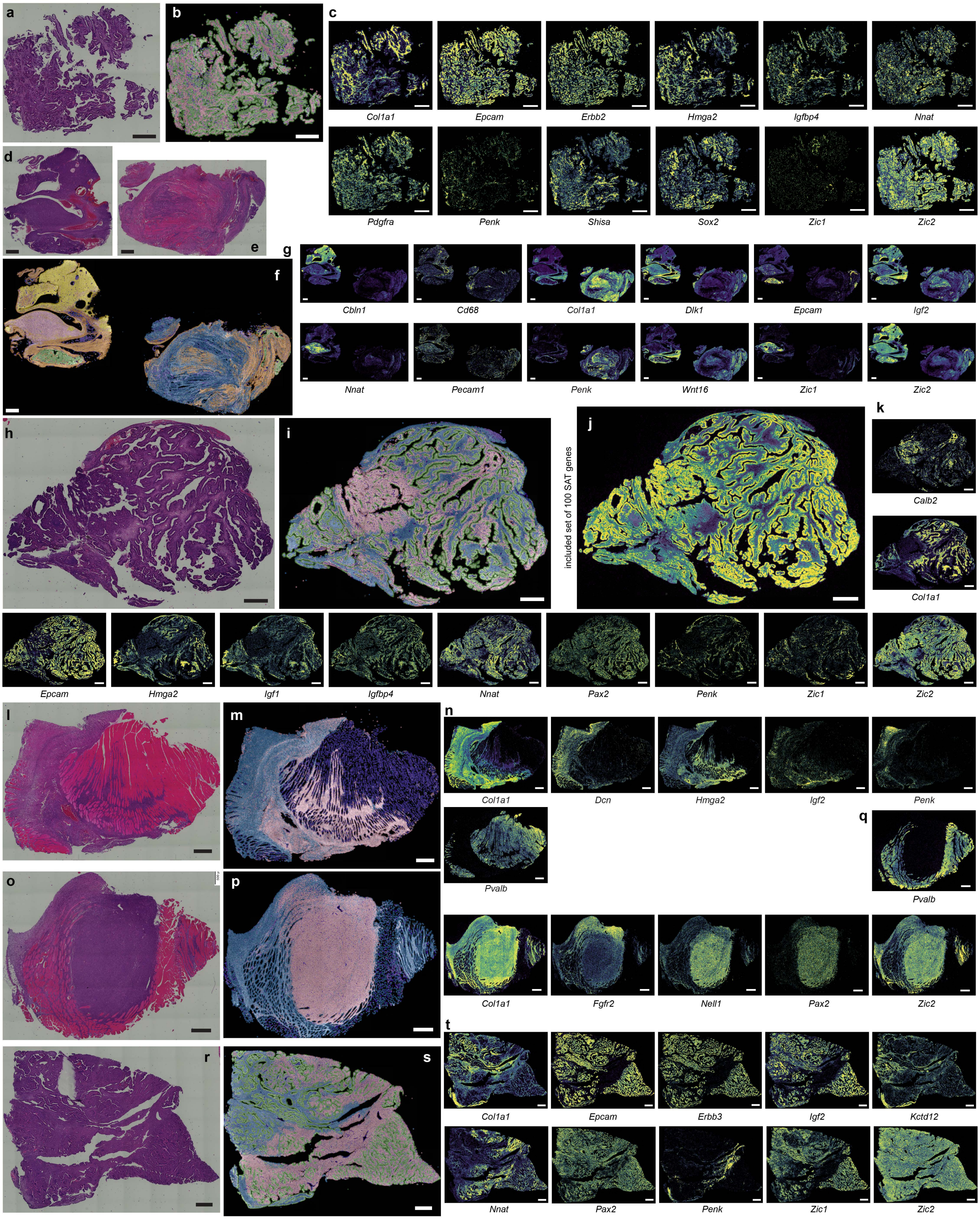

a, **d**, **e**, **h**, **l**, **o**, **r** Photomicrographs of *hSS2* mouse tumor sections stained with hematoxylin and eosin (H&E). **b**, **f**, **i**, **m**, **p**, **s** Xenium In Situ pseudo-colored spatial representation of clusters of cells: monophasic (blue), epithelial and poorly differentiated (pink), glandular epithelial cells (green), endothelial cells (purple). **c**, **g**, **j**, **k**, **n**, **q**, **t** Spatial heat maps of gene expression following Xenium In Situ. (Magnification bars, 500μm).

Supplementary Fig. 5 (associated with Figure 4): Pseudo-time analysis defines gene sets associated with reprogramming.

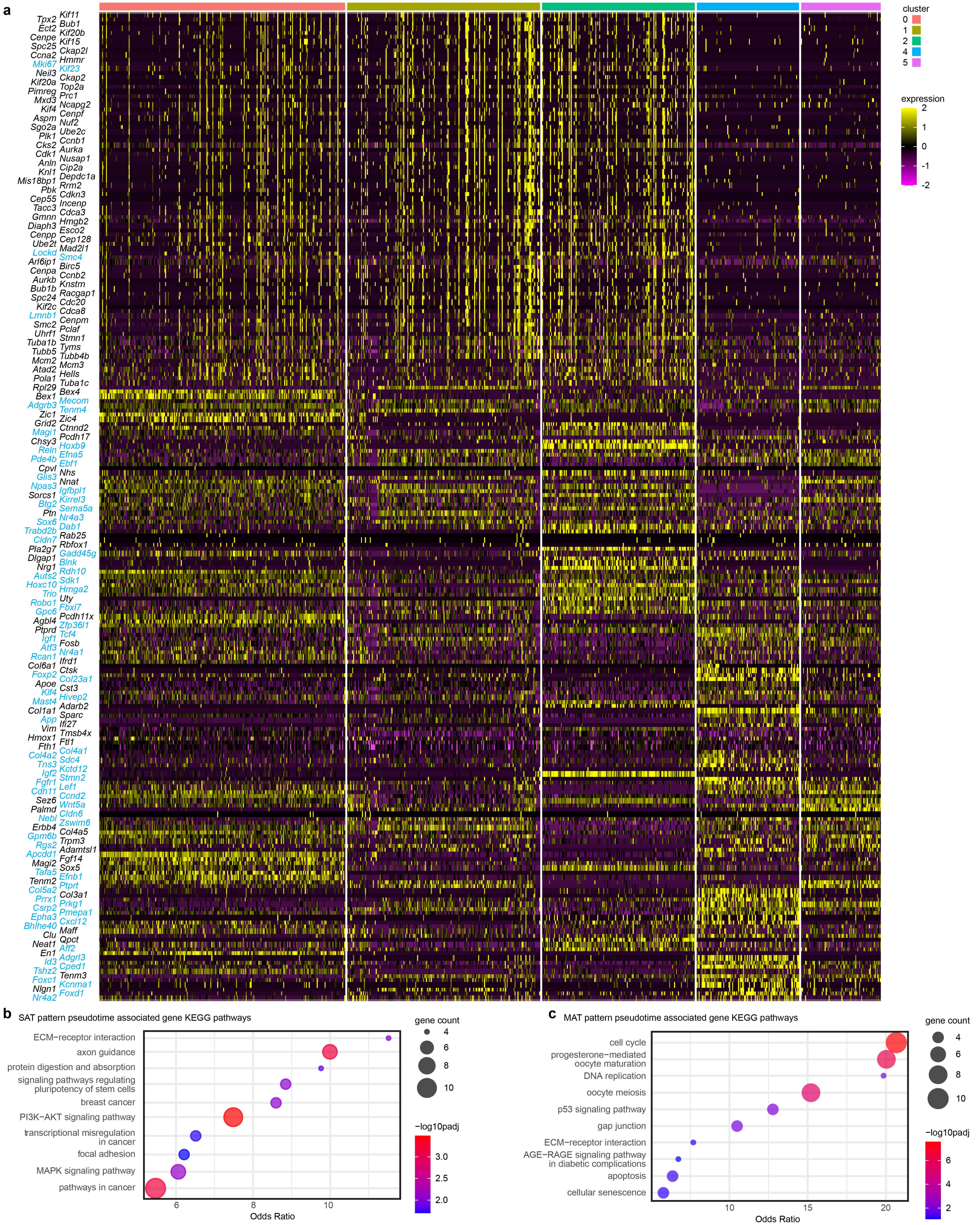

a Heatmap depicting gene expression profiles associated with pseudo-time trajectories in tumor cell populations (SAT pattern genes, cyan text; MAT pattern genes, black text). **b** KEGG pathway analysis of SAT pattern genes and **c** MAT pattern genes, each from the pseudo-time associated list.

Supplementary Fig. 6 (associated with Fig. 5). Accompanying SMARCB1 loss alters synovial sarcoma phenotypes.

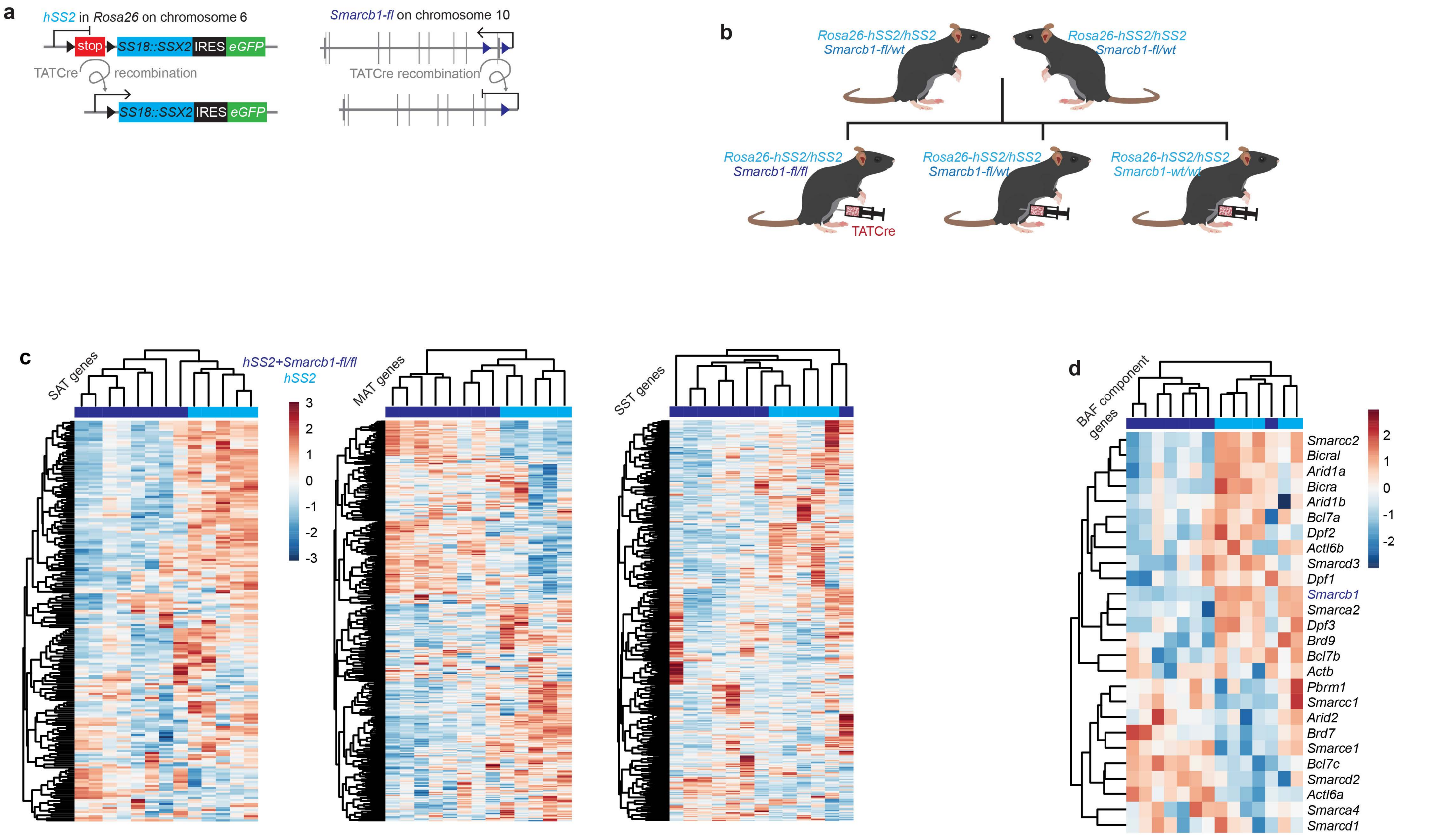

a Schematic of the *hSS2 and Smarcb1*-floxed alleles, for simultaneous expression of SS18::SSX2 and deletion of critical *Smarcb1* exons upon Cre-mediated recombination. **b** *hSS2* breeding scheme to generate littermate-controlled tumorigenesis phenotyping experiments with varied *Smarcb1* genotypes. **c** Expression heatmaps for SAT, MAT, and SST genes expressed in *hSS2;Smarcb1-wt/wt and hSS2;Smarcb1-fl/fl* tumors. **d** Expression heatmaps for BAF component genes to test the depth of loss of the conditionally targeted allele and for any compensatory changes in other components.

Supplementary Fig. 7 (associated with Fig. 6). Accompanying PBRM1 loss alters synovial sarcoma phenotypes.

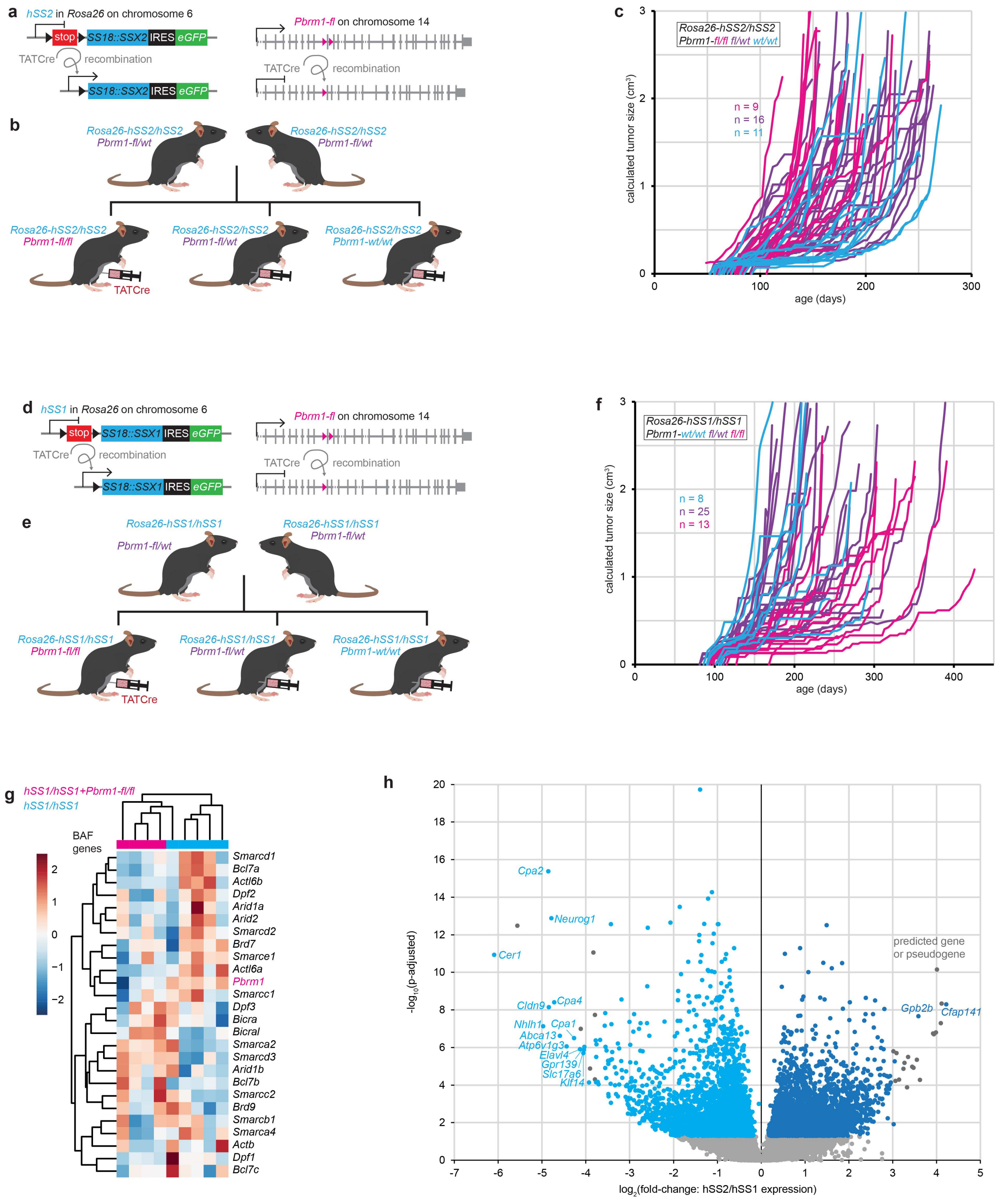

a Schematic of the *hSS2* and *Pbrm1-floxed* alleles, for simultaneous expression of SS18::SSX2 and excision of *Pbrm1* exon 11 by Cre-mediated recombination. **b** *hSS2* breeding scheme to generate littermate-controlled tumorigenesis phenotyping experiments with varied *Pbrm1* genotypes. **c** Individual tumor growth trajectories for cohorts of *hSS2* mice with varied *Pbrm1* genotypes. **d** Schematic of *hSS1* allele and the *Pbrm1-floxed* allele and the breeding scheme **e** to generate littermate-controlled tumor phenotyping experiments. **f** Individual growth curves in *hSS1* mice injected with TATCre at day 8 of life comparing littermates with varied *Pbrm1-fl* genotypes. **g** Expression heatmap for bulk RNA-seq of BAF components among *hSS1* tumors arising in mice with homozygous floxed or wildtype *Pbrm1*, demonstrating only subtle reduction in *Pbrm1* in the homozygous floxed tumors, suggesting heterozygous recombination of the floxed alleles in the cells that gave rise to these tumors. **h** Differential expression comparing hSS2 to hSS1 driven tumor transcriptomes without secondary genetic alterations shows mostly predicted and pseudogene, or spurious differences, such as pancreatic gene expression differences.

Supplementary Fig. 8 (associated with Fig. 7): CBAF component disruptions enhance synovial sarcomagenesis.

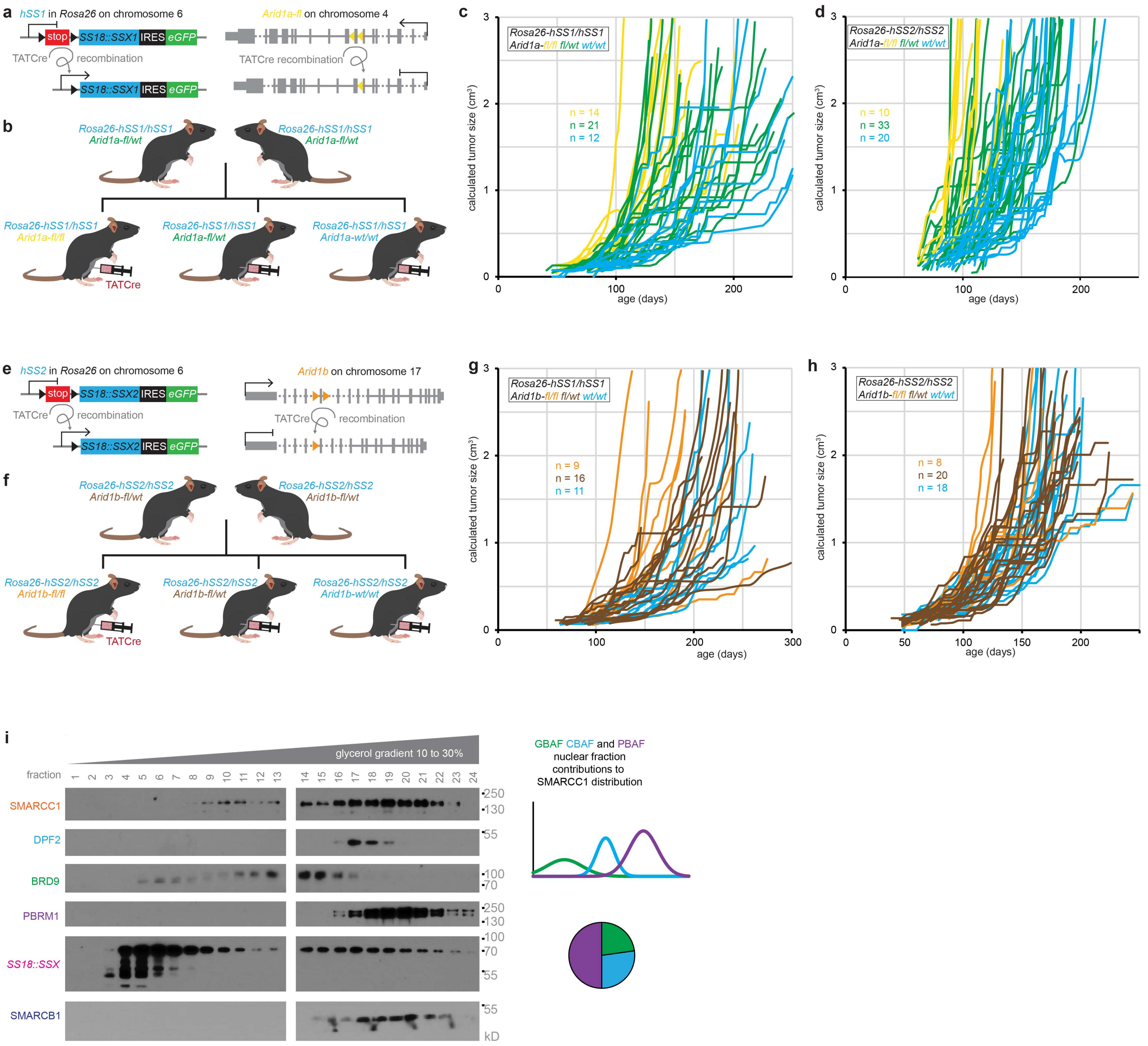

a Schematic of *hSS1* and *Arid1a-floxed* alleles, for simultaneous expression of SS18::SSX1 and excision of *Arid1a* exon 8 by Cre-mediated recombination. **b** Breeding scheme for generating littermate-controlled cohorts of *hSS1* mice bearing variable *Arid1a* genotypes. **c** Individual growth curves for *hSS1* tumors with variable *Arid1a* genotypes, as well as **d** for *hSS2* phenotyping experiments with varied *Arid1a* genotypes. **e** Schematic of *hSS2* and *Arid1b-floxed* alleles, for simultaneous expression of SS18::SSX2 and excision of *Arid1b* exon 5 by Cre-mediated recombination. **f** Breeding scheme for generating littermate-controlled cohorts of *hSS2* mice bearing variable *Arid1b* genotypes. **g** Individual growth curves for *hSS1* tumors with variable *Arid1b* genotypes, as well as **h** for *hSS2* phenotyping experiments with varied *Arid1b* genotypes. **i** Western blots of BAF components following glycerol size fractionation of nuclear extracts, showing contributions of CBAF, GBAP and PBAF to the overall SMARCC1 distribution calculated for each fraction.

Supplementary Fig. 9 (associated with Fig. 8): Unlike *Arid1a* disruption, *Pbrm1* disruption alters synovial sarcoma transcriptional features.

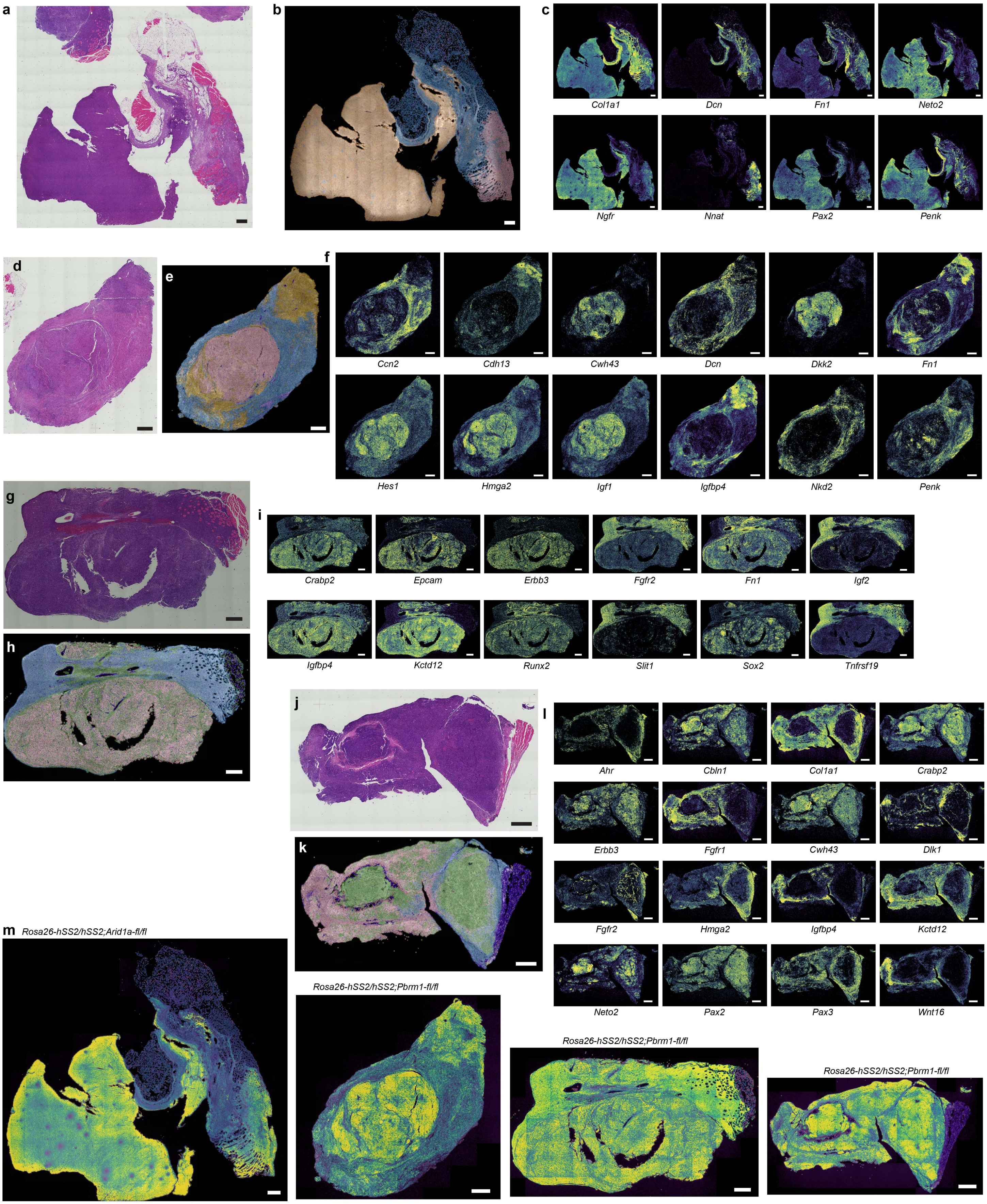

a Hematoxylin and eosin (H&E) photomicrograph of a *Rosa26-hSS2/hSS2;Arid1a-fl/fl* tumor with **b** Xenium clustering annotation and **c** expression heatmaps for individual genes. **d**, **g**, **j** H&E photomicrographs of *Rosa26-hSS2/hSS2;Pbrm1-fl/fl* tumors with Xenium clustering **e**, **h**, **k** and expression heatmaps for individual genes **f**, **i**, **l**. **m** Heatmaps for expression of a 100 SAT gene set included on the Xenium. (All magnification bars are 500μm in length).

Supplementary Fig. 10 (associated with Fig. 8): Human synovial sarcomas observations correlate with findings from mouse disruptions of *Arid1a*, *Arid1b*, *Pbrm1*, or *Smarcb1*.

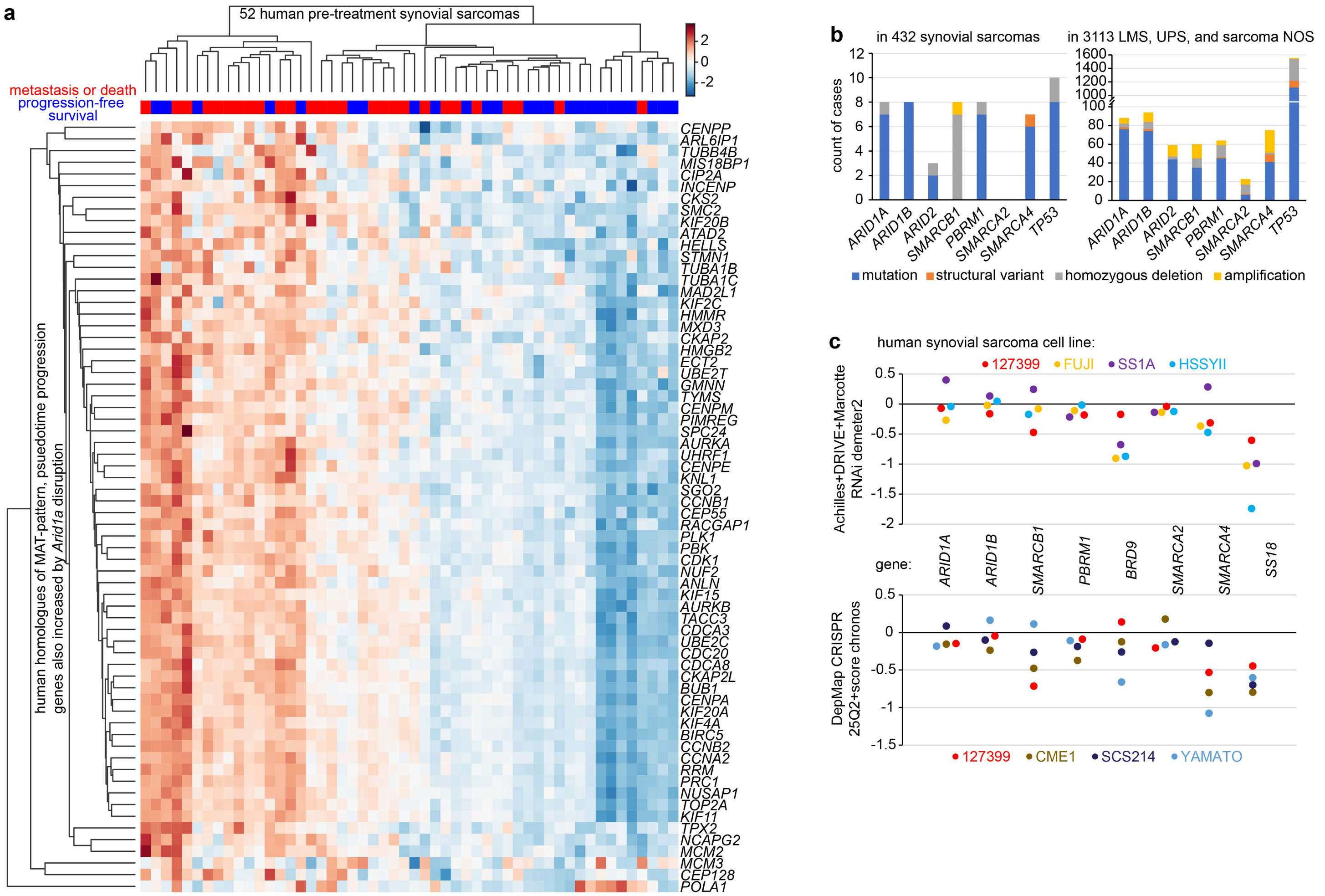

a Nonhierarchical clustering of differential expression of the human homologues of MAT-pattern pseudotime-associated genes with human SySs categorized by favorable or poor prognosis. **b** Number of cases of each identified DNA-level genomic change of the associated BAF subunit (or positive control of *TP53*) loci in human SySs or a comparison cohort of undifferentiated pleiomorphic sarcoma (UPS), leiomyosarcoma (LMS), and sarcoma not otherwise specified (NOS) cases from cBioPortal. **c** DepMap portal defined DEMETER2 scores from RNA interference (RNAi) among human SyS cell lines in the Achilles, DRIVE, and Marcotte databases and CRISPR/Cas9 chronos scores from the 25Q2 DepMAP database for BAF subunit genes.
