# Supplementary material for: Synovial sarcoma reprograms transcription by GBAF activation of polycomb targets and loss of CBAF enhancers
Source: Nat Commun. 2025 Dec 21;17:1081. doi: 10.1038/s41467-025-67841-y (PMC12852873; doi:10.1038/s41467-025-67841-y)
Supplement: Supplementary file 2 — Reporting Summary [file 41467_2025_67841_MOESM2_ESM.pdf]

nature portfolio

Corresponding author(s): *Kevin B. Jones*

Last updated by author(s): Sep 5, 2025

Reporting Summary

Nature Portfolio wishes to improve the reproducibility of the work that we publish. This form provides structure for consistency and transparency in reporting. For further information on Nature Portfolio policies, see our Editorial Policies and the Editorial Policy Checklist.

Statistics

For all statistical analyses, confirm that the following items are present in the figure legend, table legend, main text, or Methods section.

n/a	Confirmed	
☐	☒	The exact sample size (*n*) for each experimental group/condition, given as a discrete number and unit of measurement
☐	☒	A statement on whether measurements were taken from distinct samples or whether the same sample was measured repeatedly
☐	☒	The statistical test(s) used AND whether they are one- or two-sided<br>*Only common tests should be described solely by name; describe more complex techniques in the Methods section.*
☐	☒	A description of all covariates tested
☐	☒	A description of any assumptions or corrections, such as tests of normality and adjustment for multiple comparisons
☐	☒	A full description of the statistical parameters including central tendency (e.g. means) or other basic estimates (e.g. regression coefficient) AND variation (e.g. standard deviation) or associated estimates of uncertainty (e.g. confidence intervals)
☐	☒	For null hypothesis testing, the test statistic (e.g. *F*, *t*, *r*) with confidence intervals, effect sizes, degrees of freedom and *P* value noted<br>*Give P values as exact values whenever suitable.*
☒	☐	For Bayesian analysis, information on the choice of priors and Markov chain Monte Carlo settings
☒	☐	For hierarchical and complex designs, identification of the appropriate level for tests and full reporting of outcomes
☐	☒	Estimates of effect sizes (e.g. Cohen's *d*, Pearson's *r*), indicating how they were calculated

Our web collection on statistics for biologists contains articles on many of the points above.

Software and code

Policy information about availability of computer code

Data collection	*Living image software PerkinElmer IVIS system version 4.7.3.*
Data analysis	*GraphPad Prism software (version 9.0); featureCounts (version 1.6.3); CellRanger (v7.2.0) Monocle 3 (version 1.3.4); Seurat package (v5.0.3); HiC-Pro (v2.11.4); Hichipper (v0.7.0); Novoalign (Version 3.00);MACS2 (version 2.2.9); deepTools (version 3.5.6) ; STAR (2.7.11); DESeq2 version 3.11; Scanpy (v1.10.0)*

For manuscripts utilizing custom algorithms or software that are central to the research but not yet described in published literature, software must be made available to editors and reviewers. We strongly encourage code deposition in a community repository (e.g. GitHub). See the Nature Portfolio guidelines for submitting code & software for further information.

Data

Policy information about availability of data

All manuscripts must include a data availability statement. This statement should provide the following information, where applicable:

- Accession codes, unique identifiers, or web links for publicly available datasets
- A description of any restrictions on data availability
- For clinical datasets or third party data, please ensure that the statement adheres to our policy

The genomics dataset generated from this study are available at GSE269770, GSE269772, GSE269773 on the GEO.

Research involving human participants, their data, or biological material

Policy information about studies with human participants or human data. See also policy information about sex, gender (identity/presentation), and sexual orientation and race, ethnicity and racism.

Reporting on sex and gender	*N/A*
Reporting on race, ethnicity, or other socially relevant groupings	N/A
Population characteristics	*N/A*
Recruitment	*N/A*
Ethics oversight	*N/A*

Note that full information on the approval of the study protocol must also be provided in the manuscript.

Field-specific reporting

Please select the one below that is the best fit for your research. If you are not sure, read the appropriate sections before making your selection.

☒ Life sciences ☐ Behavioural & social sciences ☐ Ecological, evolutionary & environmental sciences

For a reference copy of the document with all sections, see nature.com/documents/nr-reporting-summary-flat.pdf

Life sciences study design

All studies must disclose on these points even when the disclosure is negative.

Sample size	*Sample sizes are explained throughout the manuscript. For the mouse tumorigenesis study, n=10 was the goal for each group to ensure the adequate statistical power.*
Data exclusions	*No data was excluded from analysis.*
Replication	*Mouse bearing SS18::SSX1 or SS18::SSX2 were employed as replicate for the tumorigenesis study. Technical replicates of the experiment are indicated in the figure legends where applicable.*
Randomization	*Mouse littermates were used as controls, and the sex, strain, and age were randomized according to NIH protocol.*
Blinding	*All pathology assessments involved reviewing tissue samples in a blinded manner. Unsupervised analysis was utilized for all the genomic datasets.*

Behavioural & social sciences study design

All studies must disclose on these points even when the disclosure is negative.

Study description	*N/A*
Research sample	*N/A*
Sampling strategy	*N/A*
Data collection	*N/A*
Timing	*N/A*
Data exclusions	*N/A*
Non-participation	*N/A*
Randomization	*N/A*

Ecological, evolutionary & environmental sciences study design

All studies must disclose on these points even when the disclosure is negative.

Study description	N/A
Research sample	N/A
Sampling strategy	N/A
Data collection	N/A
Timing and spatial scale	N/A
Data exclusions	N/A
Reproducibility	N/A
Randomization	N/A
Blinding	N/A

Did the study involve field work? ☐ Yes ☒ No

Field work, collection and transport

Field conditions	N/A
Location	N/A
Access & import/export	N/A
Disturbance	N/A

Reporting for specific materials, systems and methods

We require information from authors about some types of materials, experimental systems and methods used in many studies. Here, indicate whether each material, system or method listed is relevant to your study. If you are not sure if a list item applies to your research, read the appropriate section before selecting a response.

Materials & experimental systems

n/a	Involved in the study	
☐	☒	Antibodies
☒	☐	Eukaryotic cell lines
☒	☐	Palaeontology and archaeology
☐	☒	Animals and other organisms
☒	☐	Clinical data
☒	☐	Dual use research of concern
☒	☐	Plants

Methods

n/a	Involved in the study	
☐	☒	ChIP-seq
☒	☐	Flow cytometry
☒	☐	MRI-based neuroimaging

Antibodies

Antibodies used

Anti-SMARCA4 antibody (Western), Abcam, Cat# ab4081, RRID:AB_304271, Lot:GR3267188-2;
Anti-SMARCA4 antibody (ChIP), Abcam, Cat# ab110641, RRID:AB_10861578, Lot:GR150844-37;
Anti-SMARCB1 antibody (Western), Bethyl, Cat# A301-087A, RRID:AB_2191714, Lot:2;
Anti-SMARCC1 antibody (Western), Proteintech, Cat# 17722-1-AP, RRID:AB_2191987, Lot:00055895;
Anti-SMARCC1 antibody (ChIP), Invitrogen, Cat# PA5-30174, RRID:AB_2547648;
Anti-SS18 antibody (Western), Cell Signaling, Cat# 21792, RRID:AB_2728667, Lot 1;
Anti-SS18-SSX antibody (Western and ChIP), Cell Signaling, Cat# 72364, Lot 2;
Anti-PBRM1 antibody (Western), Bethyl , Cat# A301-591A, RRID:AB_1078808, Lot 3

	Anti-PBRM1 antibody (ChIP), Cell Signaling, Cat# 89123, RRID:AB_2936366, Lot 1;<br>Anti-ARIAD1A antibody (Western, ChIP), Cell Signaling, Cat# 12354, RRID:AB_2637010, Lot 6<br>Anti-BRD9 antibody (Western and ChIP), Proteintech, 24785-1-AP, Lot 00052422;<br>Anti-DPF2 antibody (Western and ChIP), Abcam, Cat# ab134942, RRID:AB_2728668;<br>H3K4me1 (ChIP), Diagenode, Cat# C15410037-50, CiteAb: pAb-037-050, Lot A1657D;<br>H3K4me3 (ChIP), Cell Signaling, Cat# 9751S, RRID:AB_2616028;<br>H3K27ac (ChIP and HiChIP), Abcam, Cat# ab4729, RRID:AB_2118291;<br>H3K27me3 (ChIP), Diagenode, Cat# C15410069, CiteAb: pAb-069-050, Lot A1811-001P;<br>H3K36me3 (ChIP), Abcam, Cat# ab9050, RRID:AB_306966, Lot A1857P;<br>H2AK119ub (ChIP), Cell Signaling, Cat# 8240T, RRID:AB_10891618;<br>Goat Anti-Rabbit IgG (H + L)-HRP Conjugate, BIO-RAD, Cat# 170-6515, RRID:AB_11125142;<br>Goat Anti-Mouse IgG (H + L)-HRP Conjugate, BIO-RAD, Cat# 170-6516, RRID:AB_11125547.<br>SOX2 (IF), Abcam, Cat# ab97959, RRID:AB_2341193<br>TLE1 (IF), Abcam, ab183742<br>Goat Anti-Rat IgG (H+L) Antibody, Alexa FluorTM 647 Conjugated (IF), Invitrogen, Cat# A-21247, RRID:AB_141778<br>Goat Anti-Rabbit IgG (H+L) Antibody, Alexa FluorTM 594 Conjugated (IF), Invitrogen, Cat# A-11012, RRID:AB_141359<br>Goat anti-Chicken IgY (H+L) Secondary Antibody, Alexa Fluor™ 488 (IF), Invitrogen, Cat# A-11039, RRID:AB_2534096<br>GFP (IF), Abcam, Cat# ab13970, RRID:AB_300798
Validation	Each antibody was validated by western, IF, or ChIP-qPCR

Eukaryotic cell lines

Policy information about cell lines and Sex and Gender in Research

Cell line source(s)	*N/A*
Authentication	*N/A*
Mycoplasma contamination	*N/A*
Commonly misidentified lines<br>(See ICLAC register)	*N/A*

Palaeontology and Archaeology

Specimen provenance	*N/A*
Specimen deposition	*N/A*
Dating methods	*N/A*

☐ Tick this box to confirm that the raw and calibrated dates are available in the paper or in Supplementary Information.

Ethics oversight	*N/A*

Note that full information on the approval of the study protocol must also be provided in the manuscript.

Animals and other research organisms

Policy information about studies involving animals; ARRIVE guidelines recommended for reporting animal research, and Sex and Gender in Research

Laboratory animals	Mus musculus was used, mixed strain of CB57BI/6-svJ aged between 2 to 6 months.
Wild animals	*No wild animals was used in the study.*
Reporting on sex	*Both male and female mice were included in each experiment.*
Field-collected samples	*No field-collected samples were included in this study.*
Ethics oversight	*All mouse experiments were conducted in accordance with the Institutional Animal Care and Use Committees at the University of Utah (Protocol: 00001493), following international legal and ethical guidelines.*

Note that full information on the approval of the study protocol must also be provided in the manuscript.

Clinical data

Policy information about clinical studies

All manuscripts should comply with the ICMJE guidelines for publication of clinical research and a completed CONSORT checklist must be included with all submissions.

Clinical trial registration	N/A
Study protocol	N/A
Data collection	N/A
Outcomes	N/A

Dual use research of concern

Policy information about dual use research of concern

Hazards

Could the accidental, deliberate or reckless misuse of agents or technologies generated in the work, or the application of information presented in the manuscript, pose a threat to:

No	Yes	
☒	☐	Public health
☒	☐	National security
☒	☐	Crops and/or livestock
☒	☐	Ecosystems
☒	☐	Any other significant area

Experiments of concern

Does the work involve any of these experiments of concern:

No	Yes	
☒	☐	Demonstrate how to render a vaccine ineffective
☒	☐	Confer resistance to therapeutically useful antibiotics or antiviral agents
☒	☐	Enhance the virulence of a pathogen or render a nonpathogen virulent
☒	☐	Increase transmissibility of a pathogen
☒	☐	Alter the host range of a pathogen
☒	☐	Enable evasion of diagnostic/detection modalities
☒	☐	Enable the weaponization of a biological agent or toxin
☒	☐	Any other potentially harmful combination of experiments and agents

Plants

Seed stocks	N/A
Novel plant genotypes	N/A
Authentication	N/A

ChIP-seq

Data deposition

☒ Confirm that both raw and final processed data have been deposited in a public database such as GEO.

☒ Confirm that you have deposited or provided access to graph files (e.g. BED files) for the called peaks.

Data access links
May remain private before publication.

RNA-seq, ChIP-seq, Hi-ChIP and scRNA-seq datasets are available on the GEO.
RNA-seq: GEO accession GSE269772, https://www.ncbi.nlm.nih.gov/geo/query/acc.cgi?acc=GSE269772,
Token :qxyrqeaipbolbef
ChIP-seq and Hi-ChIP: GEO accession GSE269770, https://www.ncbi.nlm.nih.gov/geo/query/acc.cgi?acc=GSE269770, Token: aningosshxydnev
scRNA-seq: GEO accession GSE269773, https://www.ncbi.nlm.nih.gov/geo/query/acc.cgi?acc=GSE269773, Token: inexeowytdubjaf

Files in database submission

ChIP-seq
GSM8326620 N269 DPF2
GSM8326621 N269 180
GSM8326622 N269 input
GSM8326623 N339 DPF2
GSM8326624 N339 180
GSM8326625 N339 input
GSM8326626 N302 DPF2
GSM8326627 N302 180
GSM8326628 N302 input
GSM8326629 N255 DPF2
GSM8326630 N255 180
GSM8326631 N255 input
GSM8326632 969 SS18::SSX
GSM8326633 969 BRD9
GSM8326634 969 input
GSM8326635 171 SS18::SSX
GSM8326636 171 single ARID1A
GSM8326637 171 input 1
GSM8326638 128 SS18::SSX
GSM8326639 128 input 1
GSM8326640 171 BRD9
GSM8326641 163 BRD9
GSM8326642 171 double ARID1A
GSM8326643 128 double SS18::SSX
GSM8326644 171 input 2
GSM8326645 163 input
GSM8326646 128 input 2
GSM8326647 171 double input
GSM8326648 128 double input
GSM8326649 128_T_H3K4me3
GSM8326650 128_T_H2AK119ub
GSM8326651 128_T_input
GSM8326652 163_T_H3K4me3
GSM8326653 163_T_H2AK119ub
GSM8326654 163_T_input
GSM8326655 171_T_H3K4me3
GSM8326656 171_T_H2AK119ub
GSM8326657 171_T_input
GSM8326658 857_T_H3K4me3
GSM8326659 857_T_H2AK119ub
GSM8326660 857_T_input
GSM8326661 969_T_H3K4me3
GSM8326662 969_T_H2AK119ub
GSM8326663 969_T_input
GSM8326664 Mouse_SynSa_G2_N302_MyF5_input
GSM8326665 Mouse_SynSa_B3_N302_MyF5_H3K4me1
GSM8326666 Mouse_SynSa_B4_N302_MyF5_H3K27me3
GSM8326667 Mouse_SynSa_B5_N302_MyF5_H3K36me3
GSM8326668 Mouse_SynSa_B1_N302_MyF5_H3K27ac
GSM8326669 Mouse_SynSa_C3_N308_MyF5_H3K4me1
GSM8326670 Mouse_SynSa_G3_N308_MyF5_input
GSM8326671 Mouse_SynSa_C4_N308_MyF5_H3K27me3
GSM8326672 Mouse_SynSa_C5_N308_MyF5_H3K36me3
GSM8326673 Mouse_SynSa_C1_N308_MyF5_H3K27ac
GSM8326674 Mouse_SynSa_D3_N332_1_H3K4me1
GSM8326675 Mouse_SynSa_D4_N332_1_H3K27me3
GSM8326676 Mouse_SynSa_G4_N332_1_input

GSM8326677 Mouse_SynSa_D5_N332_1_H3K36me3
GSM8326678 Mouse_SynSa_D1_N332_1_H3K27ac
GSM8326679 Mouse_SynSa_E2_N332_2_H3K4me1
GSM8326680 Mouse_SynSa_E3_N332_2_H3K27me3
GSM8326681 Mouse_SynSa_E1_N332_2_H3K27ac
GSM8326682 Mouse_SynSa_E4_N332_2_H3K36me3
GSM8326683 Mouse_SynSa_G5_N332_2_input
GSM8326684 Mouse_SynSa_F3_N292_H3K4me1
GSM8326685 Mouse_SynSa_F4_N292_H3K27me3
GSM8326686 Mouse_SynSa_F5_N292_H3K36me3
GSM8326687 Mouse_SynSa_G6_N292_input
GSM8326688 Mouse_SynSa_F1_N292_H3K27ac

Genome browser session (e.g. UCSC)

http://genome.ucsc.edu/s/Li%20Li/mouse_SS_BAF_histoneMarkers

Methodology

Replicates

SS18-SSX (ChIP): 4
PBRM1 (ChIP): 4
ARIAD1A (ChIP):2
BRD9 (ChIP): 3
DPF2 (ChIP): 4
H3K4me1 (ChIP): 5
H3K4me3 (ChIP): 5
H3K27ac (ChIP): 5
H3K27me3 (ChIP): 5
H3K36me3 (ChIP): 5
H2AK119ub (ChIP):5

Sequencing depth

2x150 bp paired-end~50M reads per sample for DPF2, PBRM1, SS18::SSX, BRD9, ARID1A. 2x50 bp paired-end~50M reads per sample for H3K4me1, H3K4me3, H3K27me3, H3K27ac, H3K36me3.

Antibodies

Anti-SS18-SSX antibody (ChIP), Cell Signaling, Cat# 72364, Lot 2;
Anti-PBRM1 antibody (ChIP), Cell Signaling, Cat# 89123, RRID:AB_2936366, Lot 1;
Anti-ARIAD1A antibody (ChIP), Cell Signaling, Cat# 12354, RRID:AB_2637010, Lot 6
Anti-BRD9 antibody (ChIP), Proteintech, 24785-1-AP, Lot 00052422;
Anti-DPF2 antibody (ChIP), Abcam, Cat# ab134942, RRID:AB_2728668;
H3K4me1 (ChIP), Diagenode, Cat# C15410037-50, CiteAb: pAb-037-050, Lot A1657D;
H3K4me3 (ChIP), Cell Signaling, Cat# 9751S, RRID:AB_2616028;
H3K27ac (ChIP and HiChIP), Abcam, Cat# ab4729, RRID:AB_2118291;
H3K27me3 (ChIP), Diagenode, Cat# C15410069, CiteAb: pAb-069-050, Lot A1811-001P;
H3K36me3 (ChIP), Abcam, Cat# ab9050, RRID:AB_306966, Lot A1857P;
H2AK119ub (ChIP), Cell Signaling, Cat# 8240T, RRID:AB_10891618.

Peak calling parameters

Reads were aligned to mm10 mouse genome version using Novoalign (Version 3.00) for paired-end reads. Peaks were called from each of the aligned bam files against input reads using MACS2 33, (version 2.2.9) with the parameters: callpeak -B --SPMR --qvalue= 1e-3 --mfold 15 100.

Data quality

All raw reads are subject to QC pipeline with FastQC package, which correct the issue of position-dependent biases ("Per base sequence quality" analysis), sequencing adapter contamination ("Overrepresented sequences" analysis), or DNA over amplification ("Sequence duplication levels" analysis). A further Quality control is performed in mapping step, to remove duplicates, low mapping quality alignments (< 30), check chromosome content and insert size (~200-300 bp). We finally Visualize mapped data in genome browsers (e.g., IGV) to manually inspect alignments and check for any anomalies or misalignments.

Software

Aligments: Novoalign (Version 3.00)
Visualization: igv (2.16.2)
Annotation: ChIPseeker (1.38.0)
Heatmaps and profile plots are performed by deepTools (3.5.6)
Peaks operation are done by bedtools(v2.31.0)

Flow Cytometry

Plots

Confirm that:

- [ ] The axis labels state the marker and fluorochrome used (e.g. CD4-FITC).
- [ ] The axis scales are clearly visible. Include numbers along axes only for bottom left plot of group (a 'group' is an analysis of identical markers).
- [ ] All plots are contour plots with outliers or pseudocolor plots.
- [ ] A numerical value for number of cells or percentage (with statistics) is provided.

Methodology

Sample preparation	N/A
Instrument	N/A
Software	N/A
Cell population abundance	N/A
Gating strategy	N/A

- [ ] Tick this box to confirm that a figure exemplifying the gating strategy is provided in the Supplementary Information.

Magnetic resonance imaging

Experimental design

Design type	N/A
Design specifications	N/A
Behavioral performance measures	N/A

Acquisition

Imaging type(s)	N/A
Field strength	N/A
Sequence & imaging parameters	N/A
Area of acquisition	N/A

Diffusion MRI ☐ Used ☒ Not used

Preprocessing

Preprocessing software	N/A
Normalization	N/A
Normalization template	N/A
Noise and artifact removal	N/A
Volume censoring	N/A

Statistical modeling & inference

Model type and settings	N/A
Effect(s) tested	N/A

Specify type of analysis: ☐ Whole brain ☐ ROI-based ☐ Both

Statistic type for inference

(See Eklund et al. 2016)

N/A

Correction

N/A

Models & analysis

n/a	Involved in the study
☒	☐ Functional and/or effective connectivity
☒	☐ Graph analysis
☒	☐ Multivariate modeling or predictive analysis

Functional and/or effective connectivity

N/A

Graph analysis

N/A

Multivariate modeling and predictive analysis

N/A
